# Concentrated Solar Combined With Hydrothermal Treatment to Unlock Lignin Graphitization Mechanisms

**DOI:** 10.1002/gch2.70115

**Published:** 2026-07-27

**Authors:** Salomé Rigollet, Elsa Weiss‐Hortala, Gilles Flamant, Ange Nzihou

**Affiliations:** ^1^ CNRS UMR 5302 Centre RAPSODEE Mines Albi Université De Toulouse Albi France; ^2^ Processes, Materials and Solar Energy Laboratory PROMES‐CNRS Font‐Romeu Odeillo France; ^3^ Andlinger Center for Energy and Environment Princeton University Princeton New Jersey USA

**Keywords:** biocarbon, biomass, concentrated solar, graphitization, hydrothermal carbonization, nanostructure

## Abstract

Biocarbons are carbon‐rich materials produced from biomass and are increasingly used as sustainable alternatives to fossil‐based materials for energy and environmental applications. Their production for commercial usages requires high temperature (above 2000°C), which calls for particular attention on energy consumption and environmental impact. Hydrothermal carbonization, selected as prestructuration step, produces small graphitic units with 6 stacked layers of 2.34 nm diameter. Further pyrolysis at 800°C increases layer length and the number of small graphitic units, giving an ideal graphitization precursor. Conventional and concentrated solar carbonization are compared in the graphitization step. At 1800°C, conventional carbonization leads to a turbostratic structure with a quality of graphitic domains characterized by low interlayer spacing (*d_002_* = 0.357 nm) and long defective graphene layers (*L_a(XRD)_* = 5.81 nm, *L_a(Raman)_* = 3.17 ± 0.46 nm). Solar carbonization at 1800°C also yields long defective graphene layers, but the structure is heterogeneous with both a turbostratic (*L_c_* = 2.41 nm, *d_002_* = 0.368 nm) and a graphitic (*L_c_* = 5.58 nm, *d_002_* = 0.345 nm) phase. Even though solar carbonization relies on an intermittent energy source, it enables higher graphene layer stacking biocarbon as compared to conventional carbonization route.

## Graphical abstract

Advanced carbon materials such as graphitic biocarbons are successfully obtained from kraft lignin. A sustainable production route is highlighted using a combination of hydrothermal carbonization and solar carbonization, leading to the production of long and well‐stacked graphene layers in the biocarbon.

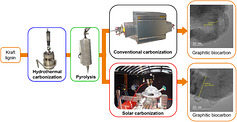

## 1 Introduction

Biocarbons are carbon‐rich materials obtained through thermal treatment of biomass or biowastes, which offer a sustainable alternative in fields where fossil‐based materials are the norm. Depending on the operating conditions and feedstock characteristics, the properties of biocarbons could vary significantly [1]. Properties such as surface functions, porosity, mineral content, and carbon structure are of particular interest for targeted applications like soil amendments [2, 3], sorbents [4, 5], biofuels [6], supercapacitors [7], or electrode batteries [8, 9]. The carbon structure is especially interesting since highly organized, graphitic biocarbons have enhanced thermal and electrical conductivity [7, 8, 10].

The thermal treatment conditions depend on the targeted properties; therefore, when graphene (2D arrangement of carbon) or graphite structures (3D stack of graphene layers) are necessary, the temperature can be quite high, exceeding 2000°C–2500°C.

Reducing the energy cost of such process is thus crucial to propose a sustainable production of graphitic biocarbon. The limitation in carbon graphitization from biomass is partially explained by the lack of prestructuration at low temperature. For soft carbons [11], Basic Structural Units (BSU), 3 stacked short (around 1 nm) graphene layers, are formed during liquid‐phase primary carbonization (200°C–550°C) [11]. Resources classified as hard carbon, such as most biomass, experience a solid phase carbonization. This is not favorable for BSU formation, and the biocarbon is hardly graphitizable. To encourage liquid‐phase carbonization, hydrothermal carbonization (HTC) is an efficient option [12].

HTC allows the conversion of biomass into hydrochar, valuable biofuels, and gas [13, 14, 15, 16]. This process mimics the long and natural coalification process, transforming organic matter into structured, carbon‐rich material. Incidentally, natural coals are widely used as graphitization precursors [17, 18]. Therefore, hydrothermal carbonization would accelerate this coalification process, resulting in suitable graphitization precursors. This pretreatment aims to obtain a carbon‐rich aromatic hydrochar. One of the macropolymers in biomass, lignin, is particularly interesting to study because of its reticulated structure and the presence of aromatic rings. During HTC of lignin, for example, phenolic and polyaromatic hydrochars are produced from solid‐solid transformations (primary char) and solid‐liquid transformations (secondary char) thanks to a sequence of reactions (hydrolysis, dehydration, polymerization, aromatization…) [19, 20, 21, 22]. This is enhanced by the HTC temperature and residence time (or high severity of the treatment), which promotes the aromatic secondary char [23]. This hydrochar composed of aromatic carbons is an ideal precursor for graphitization [12, 24].

The hydrochar itself is, however, not yet graphitic, and further thermal treatment is required to reach graphitic stage. Although HTC is a time‐consuming thermal treatment, it significantly reduces the chemical energy required for graphitization, which is of prime interest in energy‐consuming processes such as high‐temperature carbonization. Overall, the combination of processes with hydrothermal pretreatment and high temperature carbonization is beneficial in terms on energy demand [25]. The use of conventional carbonization in electrical furnaces is largely documented in the literature, with a few attempts at combining HTC and pyrolysis. It was shown recently that solar carbonization with concentrating solar systems was a sustainable alternative to produce graphitic biocarbon at high temperature [26, 27]. To the best of our knowledge, the two processes, HTC and solar carbonization, have never been combined in the literature. Concentrated solar energy has only been used to heat up hydrothermal reactors directly [28, 29] or indirectly [30].

Based on literature [12, 24, 31], the study aims at combining hydrothermal and high‐temperature carbonization to facilitate lignin graphitization below 2000°C. The objectives are to investigate the beneficial use of HTC to reduce the graphitization temperature and to assess the graphitization mechanisms from hydrochar to conventional and solar biocarbons.

## 2 Results

### 2.1 Analysis of Precursors: Hydrochar and Pyrolysis Biocarbon

The operating conditions of Hydrothermal Carbonization (HTC) are selected to promote carbon content and aromatization of the hydrochar. The reaction severity, a factor depending on temperature and reaction time, is helpful to evaluate and compare ideal operating condition [32, 33, 34]. No major structural changes were observed in the literature during lignin HTC up to 300°C, and significant aromatization only appears from 350°C (for a severity, log(𝑅0), between 9 and 10) [35, 36]. The temperature is therefore set at 350°C under autogenous pressure (18 MPa). The isotherm is held for 1 h to ensure sufficient secondary char formation [37] and to limit overall energy demand. In these conditions, the reaction severity is log(𝑅_0_) = 9.14. The resulting hydrochar matched the expectations since more than 50% of the initial carbon from kraft lignin remains in the solid hydrochar after HTC. The hydrochar is thus a carbon‐rich material (C > 80 wt.%) as expected [14, 38]. Weighing the influence of HTC in the graphitization mechanism requires to evaluate the morphology and structure of the initial hydrochar.

The particle size distribution informs on the change in particle size including agglomeration during thermal treatment, but can be influenced by the cooling method (quenching or natural cooling), the treatment temperature, the heating ramp, and the reaction time [37, 39]. The particle size distribution is determined for the initial mixture of kraft lignin in water (ratio 1:10) and for the slurry of hydrochar in aqueous media (before filtration). The results are shown in Figure S1. Initially, three mean diameters are observed in lignin. A small percentage of particles volume have a mean diameter below 0.5 µm Those small particles most probably solubilize during HTC. Another small volume of large particles with a diameter beyond 100 µm is observed, which are expected to follow solid‐liquid and solid‐solid carbonization. Most of the particles have a diameter in the range of 1–10 µm (and up to 100 µm). After HTC treatment (350°C, 18 MPa), the particle size distribution significantly evolves. Fine particles disappear in favor of larger particles. Most of the particles reach a mean diameter of a few hundreds of µm, likely representing non‐spherical aggregates formed during cooling [37]. Two other populations centered around 7 and 70 µm account for smaller aggregates. According to this measurement technique, isolated fine particles of secondary hydrochar are not observed, they aggregate at the surface of larger particles. This aggregation can be observed using Scanning Electron Microscopy (SEM).

**FIGURE 1:**
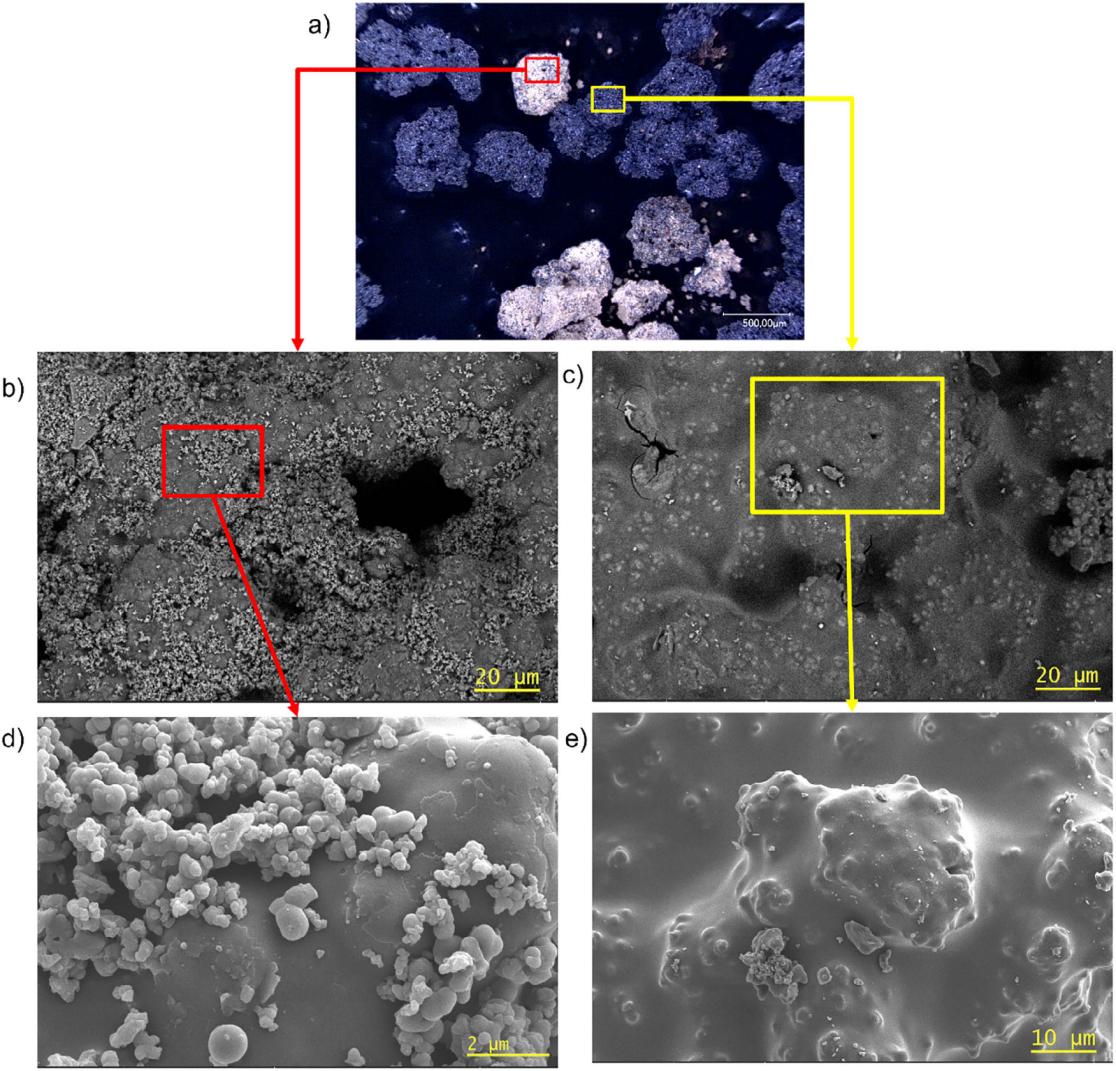
Optical image (a) and SEM image of KL‐HTC with chemical contrast at magnification 800× (b,c) and topography high‐resolution images with magnification of 10 000× and 1600×, respectively (d,e).

An optical image of hydrochar particles (KL‐HTC) is given in Figure 1a, while SEM images are displayed in Figure 1b–e. Chemical contrast images (Figure 1b,c) are obtained in low‐vacuum (1.12 mbar), while the high‐resolution images (Figure 1d,e) are taken after Pt deposition under high‐vacuum. The optical image (Figure 1a) shows random shapes of the particles, with a rough surface and a diameter of around 500 µm, agreeing with the particle size distribution. Two types of particles are identified: bright particles and darker ones, which are more abundant in the hydrochar. The chemical image (Figure 1b) of the bright particle shows numerous aggregates of different atomic mass (contrast), referring to minerals. The high‐resolution topography image (Figure 1d) reveals aggregates of more or less spherical particles with diameters of 0.1– µm. These small particles might have deposited from the liquid phase during filtration and correspond to the smallest particles (under 10 µm in particle size distribution). Carbon and oxygen are the main elements (EDXS analysis) with traces of sodium, sulfur, and potassium. This chemical analysis is not quantitative due to the surface roughness and its spatial resolution (1 µm). The darker particle has a slightly smoother surface, with small particles encapsulated under a continuous phase (Figure 1e). This particle also contains carbon and oxygen and traces of sodium and sulfur (EDXS). The EDX results are consistent with the ultimate and elemental analysis of hydrochar given in Tables S1 and S2. The carbon content is 82.49 wt.%_dry basis, ash free_ and the oxygen conent obtained by difference is 14.08 wt.%_dry basis, ash free_ (Table S1). The ICP‐OES shows high concentrations of sodium, calcium, iron, potassium, and sulfur (Table S2). The small particles under 1 µm might be assimilated to secondary hydrochar. Feedstock morphology of large particles is likely preserved and led to primary hydrochar with somewhat smooth surface decorated with agglomerated vesicles and pores (opening of vesicles due to volatilization) [35, 36, 40]. Based on the literature, secondary hydrochar is usually seen as microspheres [37, 41]. The morphology of this hydrochar therefore reveals a combination of primary and secondary chars.

Overall, the hydrothermal carbonization of lignin resulted in aggregated particles having small spherules on the surface covered in a continuous solid media, similar to the mesophase described by Brooks et al. [42].

**FIGURE 2:**
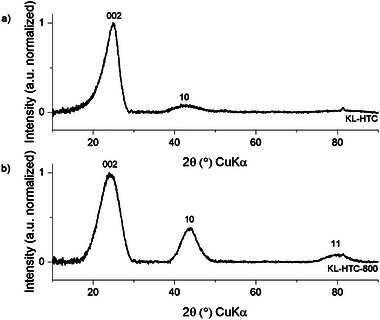
XRD patterns of (a) KL‐HTC and (b) KL‐HTC‐800.

Hydrochar carbon structure is rarely studied in literature since the thermal treatment affects the early stage of primary carbonization, where only small units (BSU) are formed. For example, the XRD pattern of hydrochar produced from coconut coir at severity log(𝑅_0_) = 6.8 (250°C, 4 h), only showed a broad *002* peak, confirming that carbon was mostly amorphous [12]. In our study, the HTC treatment is more severe and has a greater impact on the structure, as illustrated by XRD patterns. Indeed, the KL‐HTC XRD pattern (Figure 2a) shows an asymmetric *002* peak and a broad *10* peak, attesting the carbon structuration beginning. Nanotextural parameters are calculated from the XRD pattern. The interlayer spacing *d_002_* and the stacking thickness *L_c_*, obtained from the *002* peak, equal respectively to 0.377 and 1.88 nm for a mean stacking of around 6 layers. As a reminder, theoretical values of *d_002_* for graphite and turbostratic structures are 0.3354 and 0.344 nm, respectively. The interlayer spacing can be influenced by layer curvature, rotation between layers, structural defects, or intercalation, which could explain the high value of 0.377 nm obtained. The crystallite diameter *L_a(XRD)_* is 2.34 nm (obtained from *10* peak, Equation (1)). Those dimensions are slightly higher than the Basic Structural Unit (BSU) size as defined by Oberlin, 1984 [11], which is around 3 stacked layers of 1 nm diameter. BSUs seem to have formed during HTC and started to arrange in LMO (Local Molecular Oriented) domains as attested by the number of stacked layers and diameter of the crystallites. This prestructuration in BSU is promising for further carbon structuration.

The pyrolysis biocarbon pattern, KL‐HTC‐800 (Figure 2b) shows characteristic peaks of graphitic material *002*, *10* and *11* at 24.0°, 43.7°, and 79.8°, respectively. The interlayer spacing *d_002_* is equal to 0.371 nm, and the stacking thickness *L_c_* is 1.41 nm (around 4.8 layers). The crystallite mean diameter *L_a(XRD)_* is 3.68 nm. Compared to the hydrochar, the graphene layers slightly grow from the hydrochar (2.34 nm) to the pyrolysis biocarbon (3.68 nm). This is also accompanied by a slight interlayer spacing decrease from 0.377 to 0.371 nm. Although this value remains far from the graphite parameter (0.3354 nm), this indicates an improvement in the quality of graphitic domains. On the contrary, the mean crystallite thickness (and thus stacking) decreases from the hydrochar (1.88 nm, 6 stacked layers) to the intermediate biocarbon (1.41 nm, 4.8 stacked layers). Although this phenomenon is counter‐intuitive, this comes from new BSUs development (or equivalent small graphitic domains), which decreases the mean crystallite thickness. Therefore, the intermediate biocarbon has a turbostratic structure with high interlayer spacing. Pyrolysis increases the number of BSUs and the graphene layer length and decreases to a lesser extent the interlayer spacing and the stacking.

**FIGURE 3:**
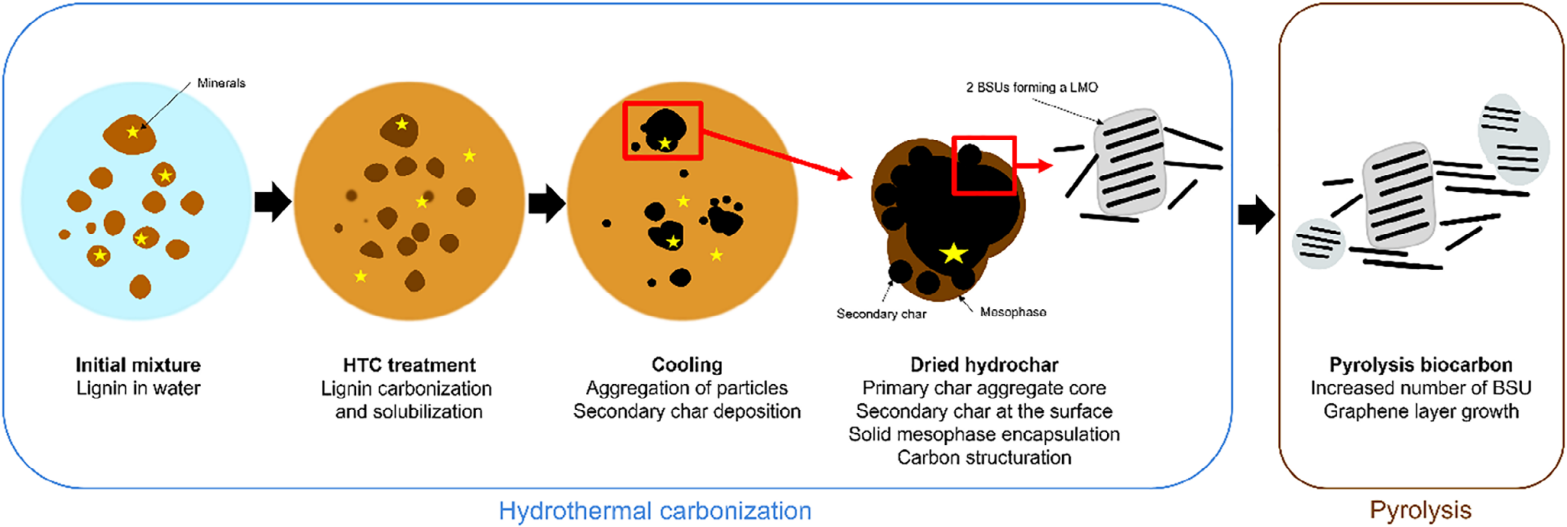
Suggested steps of hydrothermal carbonization (350°C, 1 h) followed by pyrolysis (800°C, 1 h) of kraft lignin.

Based on the literature [11, 37, 42] and the analyses described in the previous sections, a scheme of the carbon structuration main steps is proposed in Figure 3. During HTC treatment, kraft lignin is transformed through two main pathways: carbonization and solubilization. The decomposition of lignin generates a carbon rich material, the primary hydrochar (carbonization) that agglomerates during cooling into large particles of hundreds of µm wide. Part of the lignin is also dissolved in the aqueous phase (solubilization) and polymerizes into small spherules. These small particles account for the secondary hydrochar, agglomerating with primary hydrochar during cooling and filtering. The resulting dried hydrochar is a carbon‐rich material, with an irregular surface due to the mesophase encapsulation. Nevertheless, open porosity is low due to aggregates and almost spherical shapes of the particles. As developed in Boucard et al. [37], at hydrothermal conditions, the mesophase/water interface led to spherical droplets due to the minimization of Gibbs energy. This morphology is later preserved during cooling and recovered as isolated particles or agglomerates. Beyond morphology, the carbon structure of the dried hydrochar reveals BSU and small LMOs, as suggested by XRD analysis. The BSUs remain in the pyrolysis biocarbon, and their number increases as well as the length of flat graphene layers. These structures are the precursors for the graphitization step.

A similar combination of processes was proposed in Tang et al. [43] with HTC at 240°C and pyrolysis at 800°C. They also observed an improvement when using HTC pretreatment on the shape and intensity of XRD graphite peaks and thus an improvement in carbon structuration. Nanotextural values were not disclosed, but they may differ from ours due to the difference in severity of HTC pretreatment. The KL‐HTC‐800 pyrolysis biocarbon displays a favorable structure for graphitization and is thus subjected to high‐temperature graphitization in both conventional and solar processes.

### 2.2 Graphitization in Conventional and Solar Furnace

The graphitization step is done in both conventional and solar reactors at 1000°C, 1400°C, and 1800°C. In conventional carbonization, the heating ramp is low (5°C min^−1^) whereas fast carbonization is reached with solar heating (1200°C min^−1^). The heating method (bulk heating of furnace vs. surface heating) was shown to influence the relative quantity (proportion of graphitic vs. amorphous carbon) and quality (nanotextural parameters) of graphitic content [26]. Three properties are therefore studied for the final biocarbon: the structure (crystallographic state), nanotexture (quality of the graphene layer), and texture (relative orientation of graphene layers).

#### 2.2.1 Structure

**FIGURE 4:**
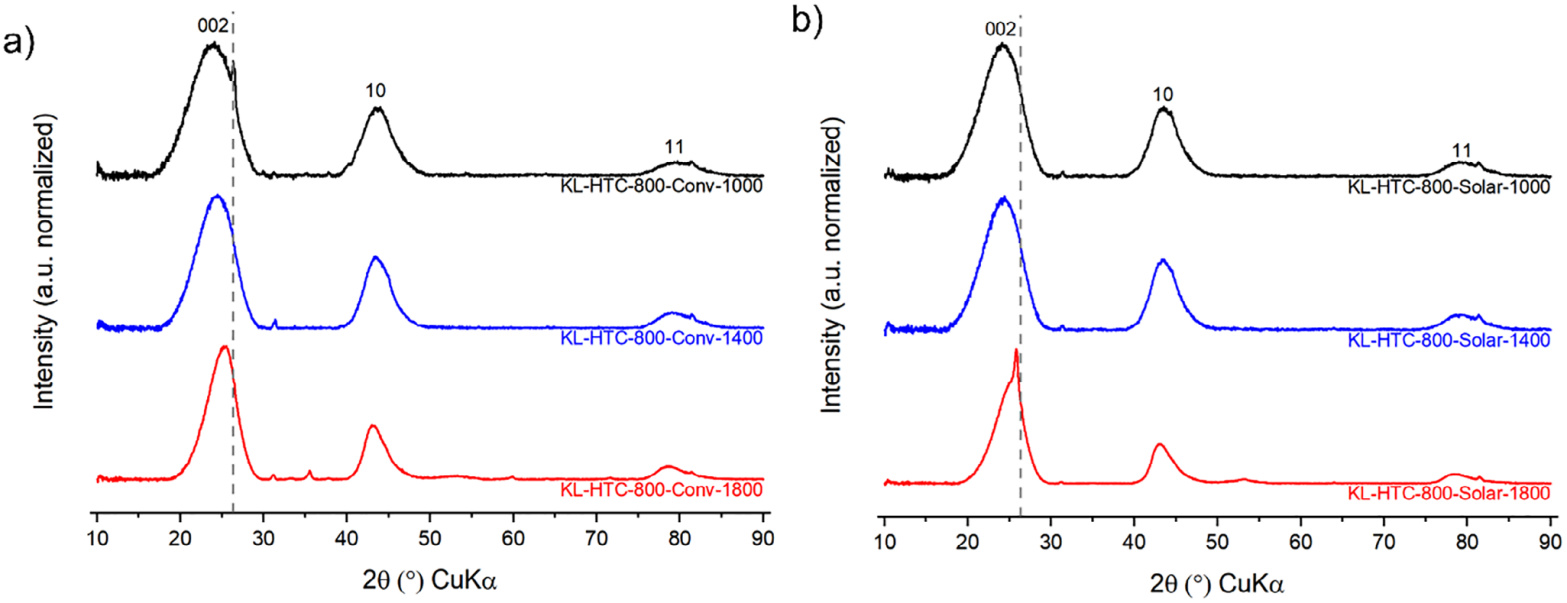
XRD patterns of (a) conventional and (b) solar carbonization at 1000°C (black), 1400°C (blue) and 1800°C (red). The dash line at 26.4° represent the position of 002 peak of hexagonal graphite.

First, the structure is investigated at the bulk scale using XRD. The XRD patterns are presented in Figure 4a for conventional and Figure 4b for solar biocarbons.

For each temperature of conventionally treated biocarbons (Figure 4a), a broad, symmetrical, and shifted 002 peak is observed. The *002* peak moves from 23.8° at 1000°C to 25.0° at 1800°C, indicating the development of graphitic structures in the biocarbon [43, 44]. The broad and symmetrical shape of the peak across all temperatures attests to an amorphous and turbostratic material. The *10* and *11* peaks of biocarbon also emerge from graphitic domains, even small. The XRD patterns confirm the high carbon composition of biocarbons with little to no mineral impurities.

The solar biocarbons (Figure 4b) obtained at 1000°C (KL‐HTC‐800‐Solar‐1000, black pattern) and 1400°C (KL‐HTC‐800‐Solar‐ 1400, blue pattern) exhibit the three characteristic peaks of graphitic biocarbons: *002*, *10*, and *11*. The peaks are broad, and in particular, the *002* peak is shifted compared to that of commercial graphite (26.4°, dashed vertical line), indicating both turbostratic and amorphous structures. The XRD pattern of solar biocarbon treated at 1800°C (KL‐HTC‐800‐Solar‐1800, red pattern) has an asymmetric *002* peak. This shoulder can be interpreted as the contribution of a turbostratic structure (phase 1, centered at 24.2° for KL‐HTC‐800‐Solar‐1800), at lower angles than graphite, based on literature [45, 46, 47], and especially in solar carbonization [26]. The sharper peak at a position closer to *002* of graphite is attributed to the graphitic contribution (phase 2, centered at 25.8° for KL‐HTC‐800‐Solar‐1800).

**FIGURE 5:**
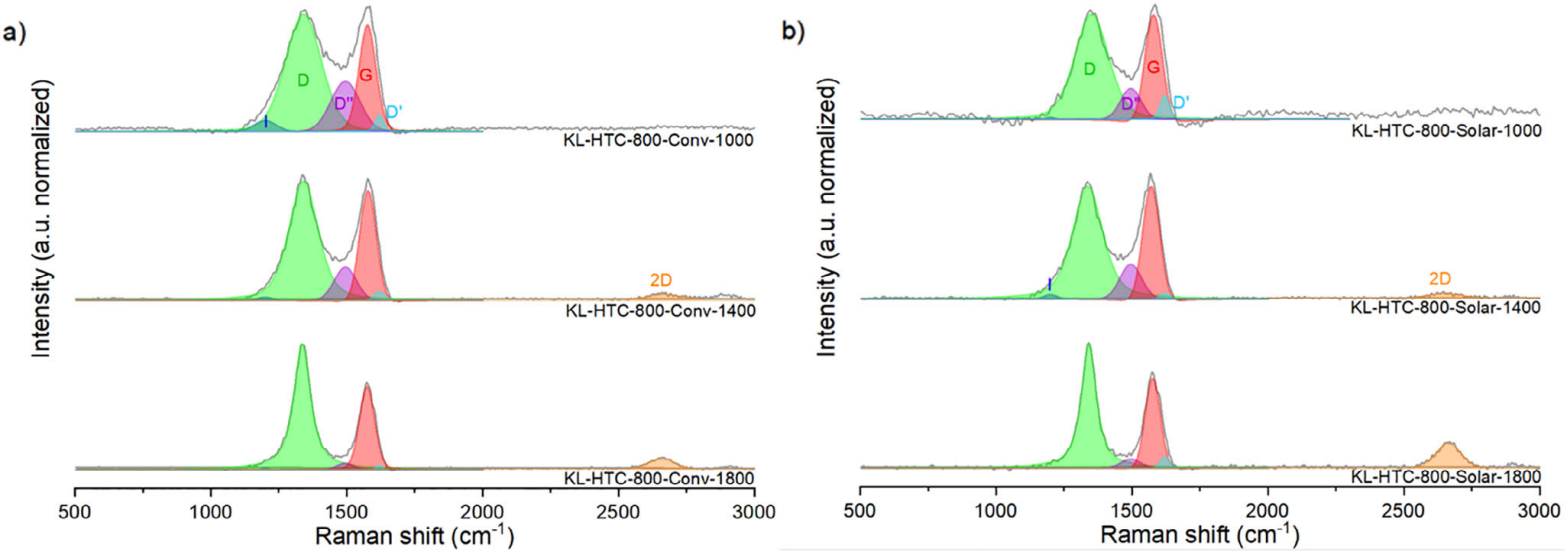
Raman spectra of (a) conventionally and (b) solar treated biocarbons at 1000°C, 1400°C, and 1800°C (top to bottom). Curve fitting is made visible with dark blue I band (fixed at 1200 cm^−1^), green D band, purple D″ band (fixed at 1495 cm^−1^), red G band, light blue D’ band (fixed at 1620 cm^−1^), and orange 2D band.

The evolution of structure with temperature is also studied at the local scale using Raman spectroscopy. A selection of Raman spectra is shown in Figure 5a. The spectra of KL‐HTC‐800‐Conv‐1000 and KL‐HTC‐800‐Conv‐1400 show large *D* (defects in graphitic structures) and *G* bands (graphitic structures) at 1340 and 1575 cm^−1^, respectively. A valley appears between those two bands, testifying of amorphous carbon (*D″* band at 1495 cm^−1^). The spectra curve‐fitting between 1200 and 1700 cm^−1^ is thus done to account for those three bands. The *2D* band around 2670 cm^−1^ is almost hindered by the background. It accounts for stacking of graphitic structures. The *2D* band relative area is very low and does not change much with temperature between 1000°C and 1400°C. The area ratio of *D″* band decreases slightly between 1000°C and 1400°C. These observations are consistent with the conclusions drawn from XRD analysis. Between 1000°C and 1400°C, the stacking is not much improved, but relative amorphous carbon decreases due to the apparition of new graphene layers. The overall structure remains turbostratic and amorphous, but the quantity of small structures increases from 1000°C to 1400°C.

A significant change is observed at 1800°C. The valley between *D* and *G* bands disappears, indicating that most of the amorphous carbon is reduced. The *2D* band is significantly more visible and its relative area goes from around 3% to 9%. This better stacking agrees with bulk scale analysis, which gave a significant higher *L_c_*. The *D* band's intensity increases with temperature as the band gets sharper. This shape at 1800°C highlights the reduction of some defects, most probably due to sp^3^ carbon consumption that otherwise contribute to a shoulder on the *D* band.

As for the conventionally treated biocarbon, the solar biocarbons (Figure 5b) obtained at 1000°C and 1400°C have large *D* and *G* bands linked with a *D″* valley band. Those spectra are thus fitted with three bands in this region. As explained previously, the *2D* band (second order) emerges as the graphitization, and especially stacking, is sufficiently developed. The *2D* band appears only from 1400°C. Although XRD analysis did not differentiate KL‐HTC‐800‐Solar‐1000 and KL‐HTC‐800‐Solar‐1400 biocarbons, the amorphous content decreases at the local scale, and the stacking is slightly improved. The amorphous content decreases with temperature up to 1800°C, while the stacking quality increases. For biocarbon KL‐HTC‐800‐Solar‐1800, the sharp *D* and *G* bands as well as an intense *2D* band highlight an improvement in carbon structuration, which can be translated in terms of nanotexture.

#### 2.2.2 Nanotexture

XRD and Raman analyses give information on the biocarbon nanotexture (Tables 1 and 2). The nanotextural parameters calculated using the Scherrer equation (Equation (1)) and Bragg law Equation (2) are summarized in Table 1 with reference to commercial graphite powder. For conventionally treated biocarbons, the interlayer spacing *d_002_* slightly decreases between 1000°C and 1400°C, accounting for a slight rearrangement of the layers. The *L_c_* (stacking thickness) and *L_a(XRD)_* (crystallite mean diameter) dimensions increase in the meantime. At these temperatures, short graphene layers continuously arise from the alignment of BSUs in the LMOs. The biocarbon produced at 1800°C (KL‐HTC‐800‐Conv‐1800) stands out a little bit more. The dimensions of stacking and diameter increase by 30% compared to 1400°C, and the number of layers goes from around 5 to 7. At this temperature, the graphene layers start to flatten and grow. The high temperature treatment initiates graphitization through the crystallite size. However, the biocarbon from conventional carbonization remains turbostratic (*d_002 =_* 0.357 nm). The effect of HTC pretreatment on lignin graphitization can be evaluated by comparing with results from Rigollet et al., 2025 [26]. For kraft lignin graphitization through conventional carbonization without HTC pretreatment, the nanotextural parameters are systematically lower at the same temperature. For example, at 1800°C without HTC pretreatment (KL‐800‐Conv‐1800), biocarbon had lower *L_c_* (1.23 nm) and *L_a(XRD)_* (3.83 nm) than the biocarbon with HTC pretreatment and conventional carbonization at 1000°C (KL‐HTC‐800‐Conv‐1000, *L_c_* = 1.29 nm and *L_a(XRD)_* = 4.44 nm). The interlayer spacing of KL‐800‐Conv‐1800 is 0.360 nm, which corresponds to around 4.4 layers stacked, and is close to that obtained for KL‐HTC‐800‐Conv‐1000 (4.5 layers). The HTC pretreatment positively influenced the nanotexture of the biocarbon by promoting BSU formation at low temperature. This further benefits the formation of a turbostratic biocarbon from 1000°C, having similar characteristics to biocarbon produced at 1800°C without pretreatment.

**TABLE 1:** Nanotextural parameters from XRD patterns of KL‐HTC‐800‐Conv and KL‐HTC‐800‐Solar. Phase 1 is the amorphous and turbostratic contribution, and phase 2 is the graphitic contribution.

	*L_c_* (nm)		*L_a(XRD)_* (nm)	*d_002_* (nm)	
Biocarbon	Phase 1	Phase 2		Phase 1	Phase 2
KL‐HTC‐800‐Conv‐1000	1.29	—	4.44	0.374	—
KL‐HTC‐800‐Conv‐1400	1.52	—	4.73	0.368	—
KL‐HTC‐800‐Conv‐1800	2.06	—	5.81	0.357	—
KL‐HTC‐800‐Solar‐1000	1.50	—	4.33	0.371	—
KL‐HTC‐800‐Solar‐1400	1.48	—	4.50	0.370	—
KL‐HTC‐800‐Solar‐1800	2.41	5.58	5.98	0.368	0.345
Commercial graphite		23.25	15.63		0.338

**TABLE 2:** Nanotextural parameters from Raman spectra of KL‐HTC‐800‐Conv and KL‐HTC‐800‐Solar.

Biocarbon	*I_D_/I_G_*	*L_a(Raman)_* (nm)	*A_I_ /A_Tot_* (%)	*A_D’’_ /A_Tot_* (%)	*A_D’_ /A_Tot_* (%)	*A_2D_ /A_Tot_* (%)
KL‐HTC‐800‐Conv‐1000	1.07	4.77 ± 0.55	1.7	14.2	1.7	2.6
KL‐HTC‐800‐Conv‐1400	1.07	4.72 ± 0.08	0.7	10.2	0.8	2.2
KL‐HTC‐800‐Conv‐1800	1.75	2.94 ± 0.50	0.1	1.2	2.7	8.8
KL‐HTC‐800‐Solar‐1000	1.05	4.83 ± 0.28	0.2	7.1	1.9	0.0
KL‐HTC‐800‐Solar‐1400	1.33	4.07 ± 1.33	1.7	9.6	1.0	4.2
KL‐HTC‐800‐Solar‐1800	1.07	5.53. ± 2.81	0.4	4.3	1.9	8.8
Commercial graphite	0.29	20.08 ± 7.87	—	—	—	—

The nanotextural dimensions for solar biocarbons obtained at 1000°C and 1400°C are similar. About 5 graphene layers (*d_002_* = 0.37 nm and *L_c_* = 1.5 nm) are stacked with about 4 nm diameter. The structure is turbostratic, and the gain of 400°C in temperature only produces more of those structures. This is seen before peak normalization where the *002* peak of KL‐HTC‐800‐Solar‐1400 is 34% more intense than that from KL‐HTC‐800‐Solar‐1000, indicating a higher number of atoms in the same arrangement [48]. At 1000°C and 1400°C, in conventional and solar carbonization post HTC, the biocarbons are characterized by a similar structure and nanotexture according to XRD analysis (Table 1). If such a turbostratic (and partially amorphous) structure is desired, it is advised to use concentrated solar energy at 1000°C to minimize the energy intake. For solar carbonization without HTC pretreatment, the nanotextural dimensions are systematically lower at 1000°C–1400°C [26]. The prestructuration using hydrothermal carbonization proved efficient to promote graphene layer formation and stacking (greater *L_a(XRD),_ L_c_* and *d_002_*).

Combination of HTC and solar carbonization at 1800°C produces a partially graphitized biocarbon (2 phases). The graphene layer flat diameter *L_a(XRD)_* increased by 33% between 1400°C and 1800°C, reaching almost 6 nm. For phase 1, the growth and flattening of graphene layers is accompanied by an increase in stacking thickness of almost 63%. The interlayer spacing is almost similar between 1400°C and phase 1 at 1800°C; the solar energy mostly impacted the number of stacked layers rather than the interlayer distance. These turbostratic characteristics (1800°C, phase 1) are similar to that from combined HTC and conventional carbonization KL‐HTC‐800‐Conv‐1800. Nevertheless, HTC + solar carbonization forms a second phase with improved characteristics compared to conventional carbonization. For Phase 2, about 17 layers are stacked with an interlayer spacing close to theoretical pure turbostratic structure (0.344 nm) [49]. For KL‐HTC‐800‐Solar‐1800, the characteristics of both phases are almost similar to that of solar carbonized lignin without HTC pretreatment (KL‐800‐Solar‐1800 [26]). The HTC pretreatment allowed a slightly higher number of stacked layers. Overall, the same structures and nanotextures are found according to XRD analysis. At 1800°C, the solar carbonization influence on structuration prevails on the contribution from HTC pretreatment. The evaluation of the graphitization in this combined process should be strengthened with analysis at the local scale.

The analysis of Raman spectra also informs on the nanotexture of the biocarbon. The average distance between two line‐defects [50] in graphene layers, *L_a(Raman)_*, is calculated from the intensity ratio of *D* and *G* bands using Tuinstra et al. [51] equation (Equation (1)) [52]. The values are summarized in Table 2 with an error on *L_a(Raman)_* obtained from the standard deviation of the 3 to 4 spectra fitted at each temperature.

For conventionally treated biocarbons, *L_a(Raman)_* decreases with increased temperature, which suggests an increase in the number of short non‐defective graphene layers. This is consistent with the *D* band intensity evolving with temperature (i.e., increase in *I_D_/I_G_* ratio), which is due to an increase in line‐defects such as edges or defective layers fusion. Simultaneously, the band area of amorphous carbon (*D″* band) and sp^3^ carbons (*I* band) significantly decreases.

The values obtained for solar biocarbon at 1000°C and 1400°C are very similar, with an average distance between defects in graphene layers of around 4 nm. This is consistent with the nanotexture characteristics from XRD where the crystallites slightly grew. Nevertheless, *L_a(Raman)_* is reduced by 16% between 1000°C and 1400°C, meaning that growing is accompanied by defects creation. For KL‐HTC‐800‐Solar‐1800 biocarbon, *L_a(Raman)_* increases to more than 5 nm. However, the very high standard deviation (± 50.9%) highlights great heterogeneity and accounts for the development of defective layers. Indeed, the high intensity of a “fine” *D* band basically refers to a certain number of defective layers. This is consistent with the two phases identified in XRD. From the solar biocarbons produced without HTC pretreatment [26], the stacking quality (reduced interlayer spacing, higher number of stacked layers) was improved at the expense of defect healing. The intense heating method applied might also create defects and not only heals them, which explains the heterogeneity in *L_a(Raman)_* at KL‐HTC‐800‐Solar‐1800.

Overall, at 1000°C and 1400°C with both processes, the biocarbon had similar nanotextural characteristics (around 5 stacked layers and 4.5 nm diameter). At 1800°C, the solar biocarbon partially reaches a graphitic structure (17 stacked layers, 6 nm diameter). The turbostratic structure from both conventional and solar biocarbon showed similar characteristics at 1800°C. Thus, solar carbonization is the smart option since the resulting structure is similar using a less energy‐demanding process. Nevertheless, a direct observation of the texture will help understanding the mechanisms involved.

#### 2.2.3 Texture

**FIGURE 6:**
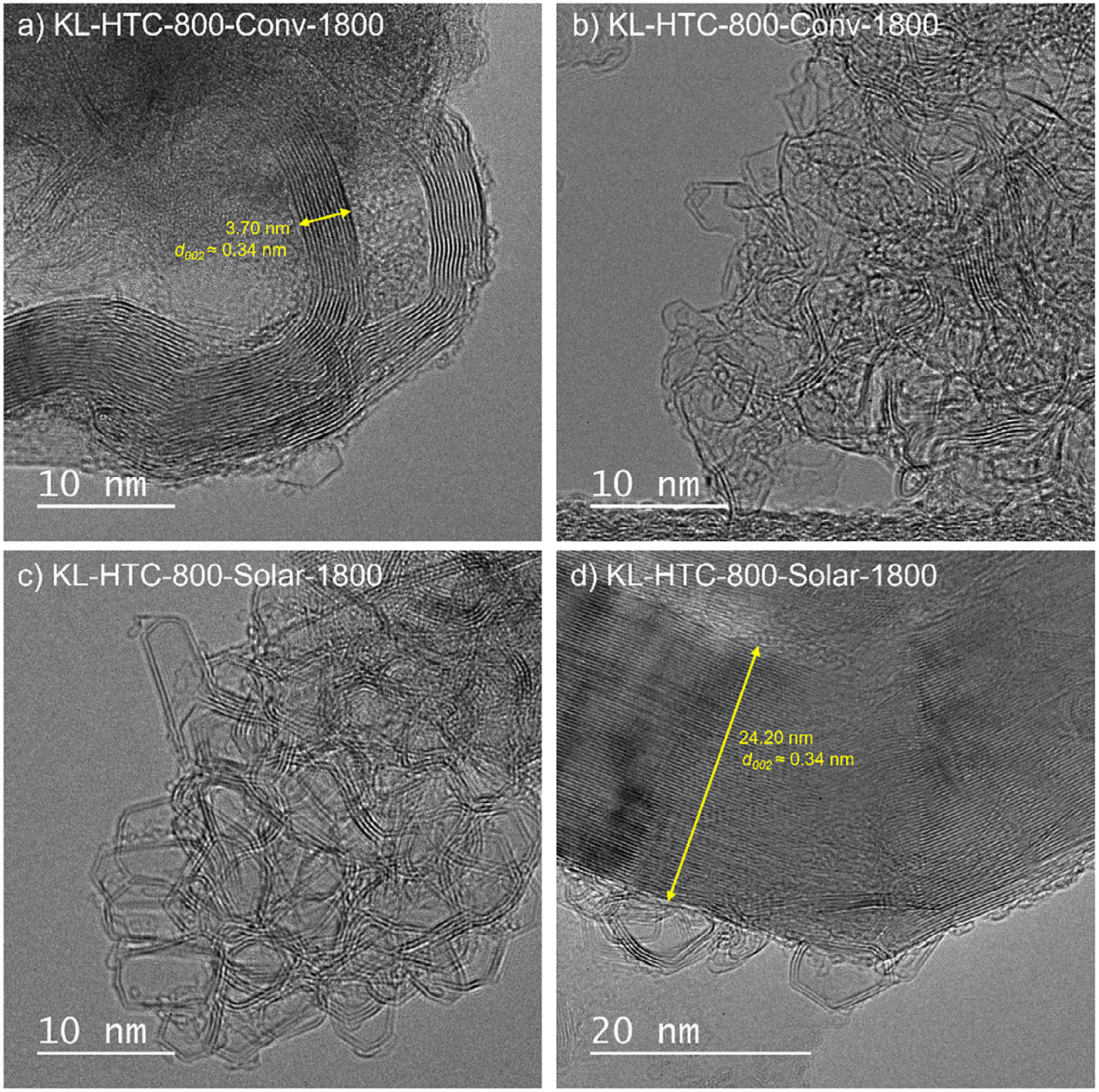
HRTEM images of KL‐HTC‐800‐Conv‐1800 (a,b magnification 500 000×) and KL‐HTC‐800‐Solar‐1800 (c magnification 500 000× and d magnification 400 000×).

The observation of graphene layers texture in the biocarbon was performed using Transmission Electron Microscopy (TEM). The high‐resolution TEM (HRTEM) images of KL‐HTC‐800‐Conv‐1800 and KL‐HTC‐800‐Solar‐1800 are presented in Figure 6.

For KL‐HTC‐800‐Conv‐1800, Figure 6a features stacks of long (> 10 nm) graphene layers with an estimated interlayer spacing of about 0.34 nm. Although the interlayer spacing approaches graphite, those stacks are composed of defective graphene layers with high curvature. Long single highly curved graphene layers, as well as stacks of a few short layers (3–6), are seen in Figure 6b. The graphene layers are aligned or have a random texture. These images illustrate the turbostratic nature of the biocarbon and agree with the conclusions drawn so far.

For the solar biocarbon (KL‐HTC‐800‐Solar‐1800, Figure 6c,d), the two phases observed in XRD also appear. The least structured phase (Phase 1) is illustrated in Figure 6c through cavity‐shaped morphology wrapped with two graphene layers. The layers are arranged in a concentric texture of inner diameter between 2 and 10 nm. This turbostratic phase coexists with a more organized phase (Figure 6d, Phase 2), containing a high number of long stacked graphene layers (from 20 to more than 80). These well‐stacked areas are slightly curved and composed of several groups with more or less defective junctions, confirming the observations done so far.

## 3 Discussion

The benefit of each combination of processes can be evaluated through two angles: the influence of the hydrothermal carbonization as a prestructuration step, and the influence of the solar carbonization as a high‐temperature treatment second.

**FIGURE 7:**
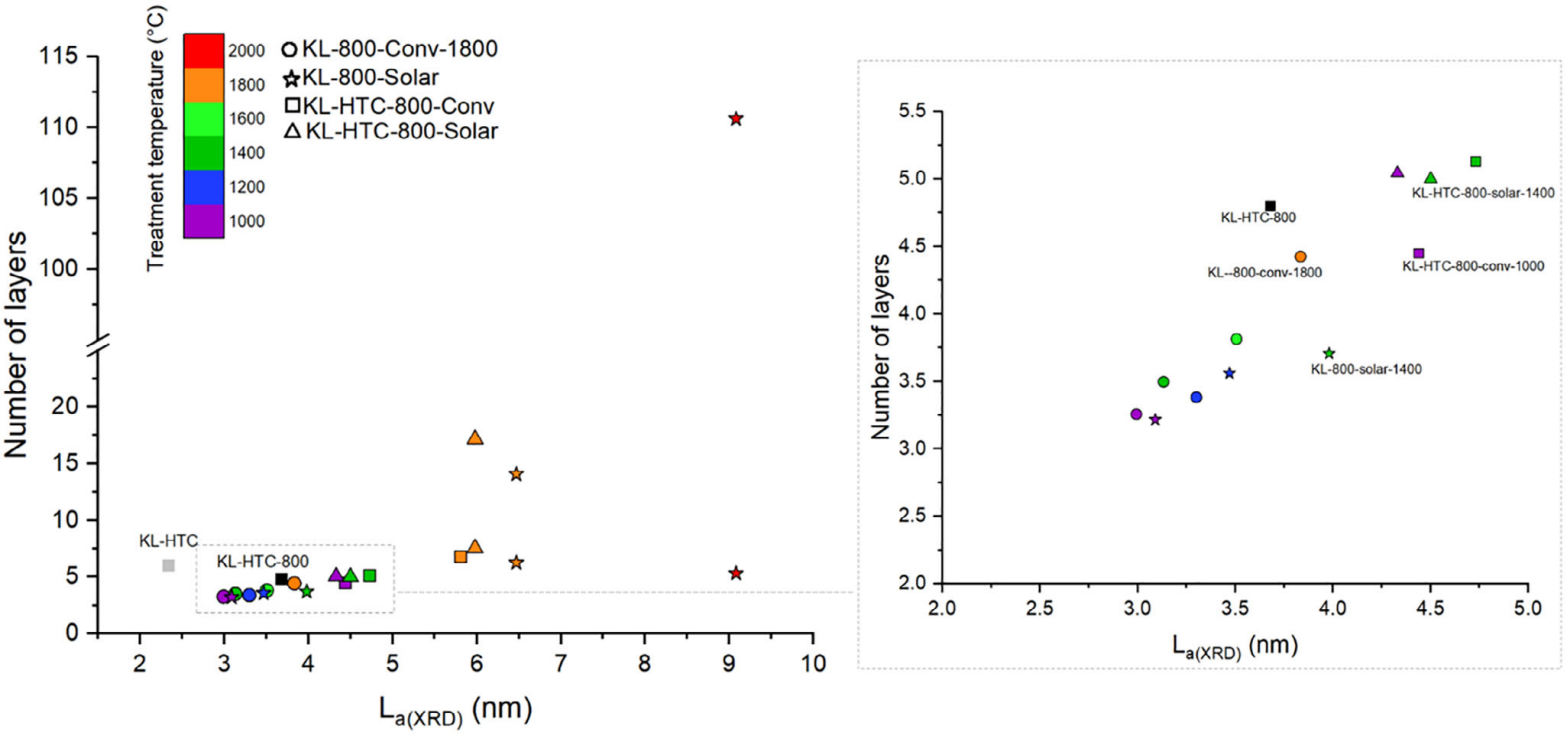
Evolution of the number of stacked layers with L_a(XRD)_ for biocarbons obtained with direct conventional (circle) or solar (star) carbonization [26] and with HTC pretreatment combined with conventional (square) or solar (triangle) carbonization at various temperatures (colored gradient) [this article].

The influence of hydrothermal pretreatment on graphitization is evidenced by comparison with previously published results on direct graphitization of kraft lignin (Figure 7) [26]. Compared to conventional carbonization, hydrothermal pretreatment as a prestructuration step proved highly efficient, since the nanotextural parameters of HTC biocarbon KL‐HTC‐800‐Conv‐1000 (purple square on Figure 7) and KL‐800‐Conv‐1800 [26] (non‐pre‐treated biocarbon, orange circle on Figure 7) are similar. Therefore, the hydrothermal prestructuration results in similar turbostratic structures at a lower graphitization energy cost. For solar biocarbons, a similar observation can be done up to 1400°C, where the quality of graphitic biocarbon is better in the case of pre‐treated biocarbons (green triangle and square on Figure 7) compared to non‐pre‐treated ones (green circle and star on Figure 7). At 1800°C however, the influence of solar carbonization prevails with no noticeable improvement from the use of hydrothermal carbonization in terms of quality and quantity of graphitic domains. A two‐phases structure (turbostratic and graphitic mix) is obtained with or without pretreatment, and it is thus advised to opt for the path with fewer steps.

Solar carbonization and its combination with HTC is thus a promising alternative to conventional carbonization, as the quality of graphitic biocarbon remains. Moreover, the energy demand is significantly lower with solar compared to conventional carbonization. The detailed calculations are given in Tables S3–S5. Overall, at lab scale, it was found that producing 1 g of graphitic biocarbon KL‐HTC‐800‐**conv**‐1800 required 17.66 kWh/g of energy, whereas producing 1 g of graphitic biocarbon KL‐HTC‐800‐**solar**‐1800 required only 0.70 kWh/g of electrical energy and an additional 3.36 kWh/g of solar energy. Moreover, the HTC pretreatment combined with pyrolysis allowed the production of a stable precursor with low mass loss during high‐temperature treatment. The initial mass required to produce 1 g of graphitic biocarbon through conventional or solar process is therefore roughly the same (around 5–6 g of initial kraft lignin). It is worth noting that these estimates were based on lab‐scale equipment for the gram‐scale production of materials. The production at larger scale with solar energy will depend on the highest target temperature and the focal zone size. A solar facility could reduce the carbon emission by half compared to an electrical one with a similar return on investment, according to Giwa et al. [53].

**FIGURE 8:**
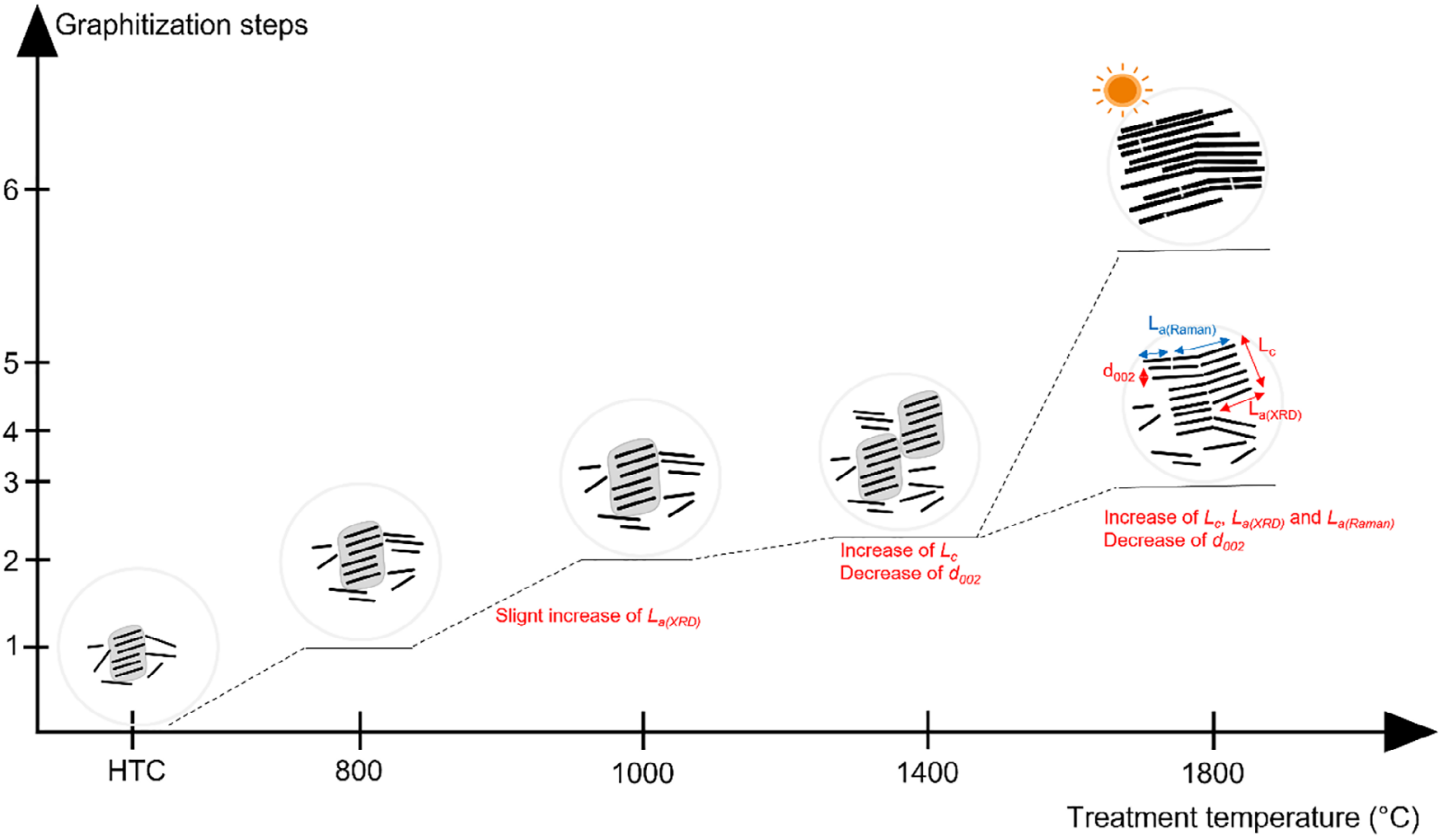
Schematic of the mechanism from hydrochar to graphitic biocarbon.

The mechanism of lignin graphitization with hydrothermal pretreatment appeared to be almost similar when using conventional or solar high‐temperature carbonization. A proposed graphitization pathway is illustrated in Figure 8.

The first step in Figure 7 corresponds to the primary carbonization, where the hydrochar is composed of a few BSU arranging in small LMOs, while the second step is the intermediate pyrolysis at 800°C, as previously illustrated in Figure 3. Hydrothermal carbonization produced a carbon‐rich material with small graphitic domains, and its combination with pyrolysis improved carbon structuration compared to direct pyrolysis. Indeed, graphitic content quality and quantity increased with HTC pretreatment compared to kraft lignin pyrolysis. The number of BSU increases, and the size of BSUs inside the LMOs slightly increases too. This agrees well with the literature [12, 43].

At 1000°C (step 3), the structure remains close to that of sample treated at 800°C, with a slight increase in crystallite diameter. At 1400°C (step 4), the number of small graphitic structures increases with a slight improvement in quality of those domains (interlayer distance decreases, stacking thickness increases). Finally, at 1800°C (step 5), the number of stacked layers increases, and the interlayer spacing decreases. The size of flat graphene layers increases together with the number of defects due to poor graphene layer junction and curvature. Step 5 illustrates the turbostratic structure with defective, curved graphene layers found in conventionally and solar treated biocarbons. Nevertheless, solar biocarbon developed a sixth step at this temperature, which corresponds to a more graphitic structure with a higher number of long, flat, stacked layers and lower interlayer spacing. This proposed mechanism is a hypothesis based on the structural and nanotextural data presented. It is also supported by the mechanism described by Zhong et al., showing a graphitization gradient with distance to the solar irradiated surface [54]. Current research focuses on in situ characterization (Pair‐Distribution‐Function, XRD, TEM) to strengthen the understanding of graphitization mechanism.

A potential application for such samples could be anode materials for Li‐ion or Na‐ion batteries. Further analysis is required to determine key parameters such as specific surface area or electrical conductivity. The nanotextural parameters, however, are promising [55].

## 4 Conclusion

Hydrothermal carbonization was successfully used as a prestructuration step for graphitization of kraft lignin. The derived carbon‐rich hydrochar reached an early stage of carbon structuration that served as a basis for graphitic phase growth (increase in stacking and growth of layers). In particular, Basic Structural Units (BSU) are formed and start to arrange in local molecular oriented domains (LMO) with around 6 short (2.34 nm diameter) graphene layers stacked with a high interlayer spacing of 0.377 nm. From those BSU, the graphene layers continue to grow and stack with pyrolysis at 800°C. The biocarbon obtained from HTC and pyrolysis is used as a graphitization precursor. The graphitization step was carried out in both conventional and concentrated solar carbonization systems. The final graphitic biocarbons produced with both methods have advantageous structural and nanotextural characteristics.

The graphitization mechanism up to 1800°C followed similar steps in both carbonization methods:

- Initial precursor (HTC + pyrolysis) contains small graphitic units (BSU).
- The crystallite diameter (*L_a(XRD)_*) increases slightly up to 1000°C, which corresponds to a growth in graphene layers. The slow growth and rearrangement of BSUs in LMOs was evident in the production of stacks of 4 short graphene layers, with *L_a(XRD)_* ∼ 4 nm and *L_a(Raman)_* ∼ 5 nm.
- At 1400°C, the quality (decrease in interlayer distance *d_002_* with stacking thickness *L_c_* increase) and quantity of small graphitic structures increase.
- At 1800°C, the conventional carbonization produced a turbostratic biocarbon (*d_002_* = 0.357 nm, *L_a(XRD)_* = 5.81 nm, *L_a(Raman)_* = 3.17 ± 0.46 nm), whereas in the solar biocarbon a turbostratic phase and a graphitic phase appeared distinctly. The turbostratic phase of HTC + solar biocarbon has similar nanotextural parameters than the HTC + conventional biocarbon, with high interlayer spacing *d_002_* of 0.368 nm and about 6 stacked graphene layers. The graphitic phase is composed of long graphene layers, as observed in TEM, with about 16 stacked layers (*d_002_* = 0.345 nm and *L_c_* = 5.58 nm).

Although the combination of processes is efficient, it is tedious. The choice of graphitization pathway should consider the targeted graphitic content and energy consumption. Thus, the comparison should be strengthened with further quantification of the different phases. Further research should focus on better quantifying the carbon phases using techniques such as XPS, pair distribution function, in situ characterizations, and NMR to improve the description of the mechanisms and better align biocarbons with their final applications. Other thermoconversion processes could also be considered, such as flash Joule heating and laser graphitization, which might be comparable to solar graphitization in terms of performance.

## 5 Experimental Section

### 5.1 Precursor Production

Kraft lignin (Sigma–Aldrich CAS: 8068‐05‐1) was pre‐treated by hydrothermal carbonization. Kraft lignin was placed in water (ratio of biomass to water of 1:10) in a stainless steel 500 mL stirred autoclave. The HTC process was carried out at 350°C, 180 bars (autogenous pressure), the heating ramp was 10°C min^−1^ and the temperature and pressure were held for 1 h. The resulting slurry was filtered, and the solid residue, the hydrochar, was dried at 105°C. The hydrochar yield reached 30% of the initial mass of kraft lignin, and the hydrochar was composed of more than 82.49 ± 1.20 wt.% (dry basis, ash free) of carbon.

The hydrochar was then pyrolyzed at 800°C in a vertical tubular furnace (Carbolite tubular furnace) for 1 h with a heating ramp of 5°C min^−1^ and under N_2_ flow. The resulting pyrolysis biocarbon had a high carbon content (89.68 ± 0.62 wt.% dry basis ash free) and low ash content (2.69 wt.%). This step was intended to prevent tar release into the laboratory graphitization furnaces.

### 5.2 Graphitic Biocarbon Production

The pyrolysis biocarbon was then carbonized at high temperature, either in a conventional electrical furnace or at the focus of a solar furnace.

The conventional electrical furnace (Nabertherm RHTH 80/300/18) was operated with a heating ramp of 5°C min^−1^ and using N_2_ gas flow rate of 6 L.min^−1^ (350 L h^−1^). A crucible containing about 5 g of sample was inserted in the heating zone of the tubular furnace, and then heated up to 1000°C, 1400°C, or 1800°C for 1 h isotherm.

The solar furnace was composed of a tracking heliostat reflecting the incident solar flux vertically toward a parabola, which concentrates the solar flux onto the sample placed in a N_2_ swiped transparent reactor. The complete system is described elsewhere [26]. A solar‐blind optical pyrometer (KLEIBER monochromatic at 5.2 µm) and a proportional‐integral‐derivative (PID) controller were used to monitor and adjust the temperature of the sample at the focus point by adjusting the incoming solar flux with shutters. Before the experiment, the emissivity of the pyrolysis biocarbon have been measured (Surface Optics Corporation Hemispheral Directional Reflectance SOC‐100 HDR reflectometer coupled with an IS50 FTIR spectrophotometer operating in the range from 1.25 to 25 µm). The emissivity of KL‐HTC‐800 measured at 5.2 𝜇m (wavelength of the pyrometer) was 0.944. Similarly to conventional carbonization, the targeted temperatures (1000°C, 1400°C, and 1800°C) were held for 1 h. The heating ramp was 1200°C min^−1^. The solid yield was between 87% (1000°C and 1400°C) and 82% (1800°C).

The biocarbons are named with the initial feedstock (KL = kraft lignin), the HTC pretreatment, the 800°C pyrolysis treatment, the type of high temperature treatment (conventional or solar), and the final temperature reached. For example, KL‐HTC‐800‐solar‐1800 is a biocarbon of kraft lignin pre‐treated with hydrothermal carbonization before pyrolysis at 800°C and solar carbonization at 1800°C.

A commercial graphite (ChemPur CAS: 7782‐42‐5) was used as a reference for its graphitic structure and texture.

**FIGURE 9:**
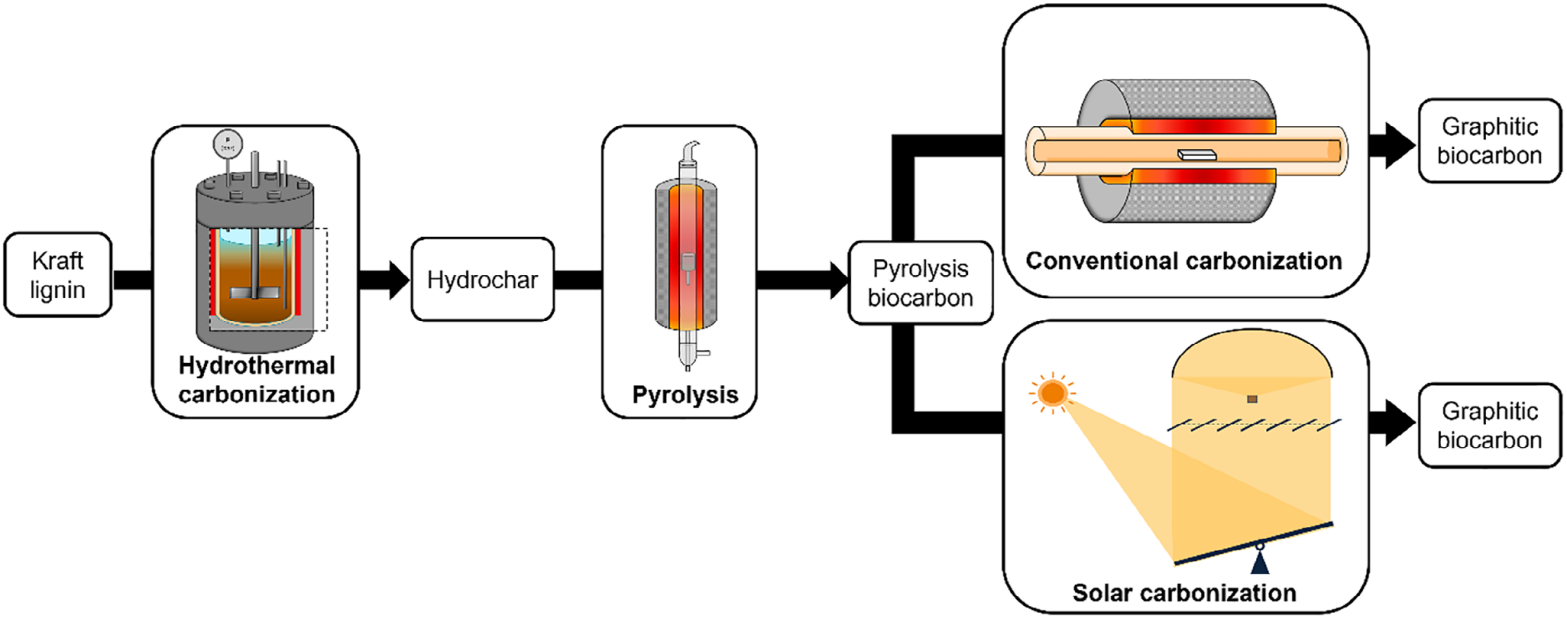
Schematic of combined processes with HTC pretreatment and high temperature treatment in solar and conventional carbonization.

The different production steps from kraft lignin to graphitic biocarbon are illustrated in Figure 9.

### 5.3 Characterization

The hydrochar's properties were first assessed. The particle size distribution was directly determined using Mastersizer 3000 Malvern for the initial mixture of lignin in water and for the slurry of hydrochar in aqueous media (before filtration) obtained after hydrothermal carbonization. The morphology and composition of hydrochar particles (after filtration and drying) were then observed at local scale using both optical microscope (digital microscope Keyence VHX1000E) and a scanning electron microscope (SEM Quattro S, Thermo Scientific). For SEM imagining, the sample was either analyzed in chemical contrast at low vacuum (1.12 mbar, 8.00 kV) or after platinum metallization for high resolution topography imaging (5.00 kV). The SEM was coupled with Energy‐dispersive X‐ray spectroscopy (EDXS) for local elemental analysis (EDAX probe, APEX software). The ultimate analysis was done using CHNO—Flash 2000 (Thermo Fisher Scientific). The elemental analysis of hydrochar was done using Inductively Coupled Plasma Optical Emission Spectrometry (ICP‐OES Horiba Ultima 2).

The biocarbons were then characterized at different observation scales. At bulk scale, the structure and nanotexture of the biocarbons were investigating using X‐ray diffraction. The diffraction patterns were obtained using an Empyrean series 3 PANanalytical X‐ray diffractometer working at 45 kV and 40 mA. The instrument was set on a Bragg‐Brentano geometry with the *CuKα* source (λ = 0.1542 nm) and the detector moving at the same speed around a fixed, rotating sample. Pattern was recorded between 10° and 90° in 2θ with a step of 0.03° and a scan speed of 0.02° s^−1^. Biocarbon patterns exhibit three characteristic peaks of hexagonal graphite structure corresponding to the planes: *002* (fitted with one or two pseudo‐Voigt curves), *10l* (fitted with a Breit‐Wigner‐Fano curve), and *11l* (Breit‐Wigner‐Fano) at approximately 24°, 44°, and 80° respectively. Nanotextural parameters can be extracted from these patterns: the in‐plane diameter, *L_a(XRD)_*, from the *10l* peak (Sherrer equation, Equation (1)), the stacking height, *L_c_*, from the *002* peak (Sherrer equation, Equation (1)), and the interlayer distance, *d_002_*, from the *002* peak (Bragg equation, Equation (2))

(1) $$L_{a,c}=\frac{K\times \lambda }{\sqrt{\beta^{2}-s^{2}}\times \cos\theta }\;$$

(2) $$d_{002}=\frac{\lambda }{2\times \sin\theta }\;$$

*λ* is the radiation wavelength in nm and *θ* the relevant Bragg angle. The correcting factor *K* is equal to 1.84 for *L_a(XRD)_* and 0.89 for *L_c_* [56, 57]. *β* and *s* are respectively the full width at half maximum of the relevant peak and standard specimen (silica, used for instrument broadening adjustment). *L_a(XRD)_*, *L_c_*, and *d_002_* are in nm.

At local scale, Raman spectroscopy also informs on the structure and nanotexture of biocarbon. The analysis was done on two zones per biocarbon, representing 900 spectra per zone. Finally, a cluster function gives a maximum of 2 mean spectra per zone. A Raman confocal microscope (WITec Alpha 300R) equipped with a 532 nm/ 2.33 eV laser was used. Six bands can be observed in biocarbon Raman spectra:

- *I* band at 1200 cm^−1^ for defects in graphitic structure, sp^3^ carbon (fitted with gaussian curve),
- *D* band at 1350 cm^−1^ for defects in graphitic structure (Pseudo‐Voigt),
- *D″* band at 1495 cm^−1^ for amorphous carbon (gaussian),
- *G* band at 1580 cm^−1^ for graphitic structure (Pseudo‐Voigt),
- *D’* band at 1620 cm^−1^ for defects in graphitic structure, C─H bonds (gaussian),
- *2D* band at 2700 cm^−1^ for well‐stacked graphitic structures (gaussian).

*L_a(Raman)_*, the length of graphene layers (distance between two in‐plane defects), is calculated using Equation (3) [51, 52, 58].

(3) $$L_{a(\mathrm{Raman})}=\frac{4.4}{I_{D}/I_{G}}{\left(\frac{2.41}{E_{L}}\right)}^{\alpha }$$

With *L_a(Raman)_* in nm, the ratio of *D* and *G* band intensities *I_D_/I_G_*, the laser energy *E_L_* in eV, and *α* the sample correction (not calculated, graphite value of α = 4 selected).

The texture of the biocarbon was observed using Transmission Electron Microscopy (TEM) in Scanning mode (STEM, 200 kV) or high‐resolution (HRTEM, 80 kV) using the JEOL JEM‐ARM200F Cold FEG microscope with probe Cs corrected.

## Funding

This work was supported by the French program “Investments for the future” (“Investissements d'Avenir”) managed by the National Agency for Research (ANR) under contract ANR‐10‐LABX‐22‐01 (Labex SOLSTICE).

## Conflicts of Interest

The authors declare no conflicts of interest.

## Supporting information




**Supporting File**: gch270115‐sup‐0001‐SuppMat.docx.

## Data Availability

The data that support the findings of this study are available from the corresponding author upon reasonable request.
