# Supplementary material for: Concentrated Solar Combined With Hydrothermal Treatment to Unlock Lignin Graphitization Mechanisms
Source: Glob Chall. 2026 Jul 27;10(7):e70115. doi: 10.1002/gch2.70115 (PMC13403054; doi:10.1002/gch2.70115)
Supplement: Supplementary file 1 — Supporting File: gch270115‐sup‐0001‐SuppMat.docx. [file GCH2-10-e70115-s001.docx]

Concentrated solar combined with hydrothermal treatment to unlock lignin graphitization mechanisms

**Supplementary materials**

**
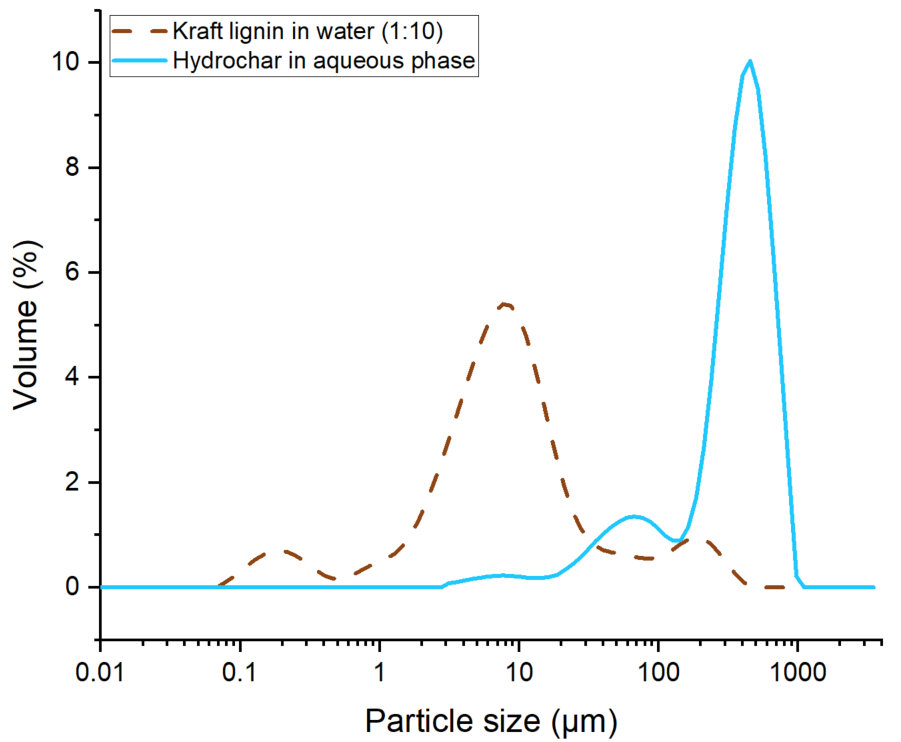
**

**Figure S1.** Particle size analysis of initial mixture of kraft lignin in water (dashed brown curve) and final hydrochar in aqueous phase (blue curve)

**Table S1.** Ultimate analysis of hydrochar (O calculated by difference)

|  | wt% dry basis, ash free |
| --- | --- |
| C | 82.49 ± 1.20 |
| H | 5.56 ± 0.04 |
| N | 0.18 ± 0.00 |
| O (by difference) | 11.17 ± 1.24 |

**Table S2.** Mineral composition of hydrochar

| Elements in mg.kg-1 | |
| --- | --- |
| Al | 712 |
| Ca | 2455 |
| Fe | 2440 |
| K | 2081 |
| Mg | 986 |
| Mn | 175 |
| Na | 12850 |
| S | 1286 |
| Si | 836 |

**Energy consumption estimation**

Energy consumption is directly estimated from the experimental reactor. It is worth noting that lab-scale reactors have a large volume compared to the feedstock mass, and finally the biocarbon mass. Therefore, the values estimated from lab furnace electrical consumption will certainly be overestimated compared to industrial data in the literature.

Four reactors consume electrical energy:

- HTC reactor: instantaneous power recorded by the instrument.
- Drying oven for hydrochar working at 105 °C: since drying oven works all day long, the experimental estimation is quite challenging and the value of 1.56 * 10^-2^ kWh to dry 10g of biomass in 12h is taken from the literature [Cusenza et al., Sustainable Production and Consumption, vol 28, pp. 866-876, 2021].
- Pyrolysis tubular furnace working at 800°C: gross estimation using the nominal power of the furnace and heating ramp duration.
- Carbonization tubular furnace (up to 1800 °C): gross estimation using the nominal power of the furnace and heating ramp duration.

The solar energy is experimentally evaluated from the direct nominal irradiance (DNI), the shutters aperture and the irradiated surface S using Equation S1.

$E\left( solar \right)=DNI\times{\%}_{aperture}\times S\times t$ (S1)

The energy E(solar) is expressed in kWh, DNI corresponds to the Direct Nominal Irradiance in W.m^-2^, the aperture of shutters %_aperture_ regulates the energy received by the sample and therefore the temperature reached and finally S and t are respectively the concentrator’s surface (1.8 m²) and the experiment duration time (in h).

The calculations are then adjusted to produce 1 g of final graphenic biocarbon. The gram scale is selected since the estimations are made from lab-scale equipment that are not designed for larger scale production.

About 30 g of kraft lignin is mixed with water (biomass:water ratio of 1:10) and introduced into the HTC reactor. The mixture is heated up to 350 °C at 10°C.min^-1^ followed by an isotherm of 1 h. After cooling, the wet hydrochar is dried overnight at 105 °C. The pretreatment produces about 9.8 g of dry hydrochar (solid yield of 32.68 ± 1.84 wt.%). Then following pyrolysis step at 800 °C (5°C.min^-1^, 1 h isotherm) results in a solid yield of 64.9 ± 0.82 wt.% from hydrochar to pyrolyzed biocarbon.

HTC equipment records the instantaneous electrical power over the experiments and equals 2.96 ± 0.13 kWh (integration of curves from 4 batch experiments). Energy related to drying the hydrochar is set at 1.56 * 10^-2^ kWh. Overall, the electrical energy consumption equals 0.30 kWh/g of hydrochar. Overall, the electrical energy consumption equals 0.30 kWh/g of hydrochar (KL-HTC).

During pyrolysis at 800 °C, the heating ramp lasts for 2.6 hours with a nominal power of the furnace of 3 kW which corresponds to 7.75 kWh. The initial hydrochar mass in this furnace is 30 g which results in 19.47 g of pyrolyzed hydrochar (KL-HTC-800) that is to say an energy consumption of 0.40 kWh/g of pyrolyzed hydrochar.

This precursor is then used for graphitization in both conventional and solar carbonization. The temperature and carbonization method influence the yield and thus the energy consumption. The conventional carbonization furnace has a nominal power of 9.2 kW and 4 g of the precursor KL-HTC-800 are introduced in the furnace. **Table S3** sums up the different parameters of high-temperature biocarbon production and their energy consumption.

**Table S3.** Electrical energy consumption calculation for high temperature conventional carbonization

| Biocarbon | Temperature (°C) | Heating ramp time (h) | Yield (%) | Electrical energy (KWh/g) |
| --- | --- | --- | --- | --- |
| KL-HTC-800-conv-1000 | 1000 | 3.3 | 88.2 | 8.60 |
| KL-HTC-800-conv-1400 | 1400 | 4.6 | 95.0 | 11.13 |
| KL-HTC-800-conv-1800 | 1800 | 5.9 | 80.0 | 16.96 |

Similarly, for solar energy the main parameters are summed up in Table S4 and an average initial mass of 400 mg is considered.

**Table S4.** Solar energy consumption calculation for high temperature solar carbonization

| Biocarbon | Temperature  (°C) | Effective heating time (h) | Yield (%) | Aperture (%) | DNI (W.m^-2^) | Solar energy  (KWh/g) |
| --- | --- | --- | --- | --- | --- | --- |
| KL-HTC-800-solar-1000 | 1000 | 1.01 | 86.8 | 16.4 | 929 | 0.79 |
| KL-HTC-800-solar-1400 | 1400 | 1.02 | 87.4 | 34.8 | 962 | 1.76 |
| KL-HTC-800-solar-1800 | 1800 | 1.03 | 82.3 | 59.3 | 1013 | 3.38 |

Finally, the mass and energy consumption for the production of 1 g of each sample are detailed in **Table S5**.

**Table S5.** Initial mass and energy required to produce 1 g of biocarbon through various process conditions

| Biocarbon | Initial mass  (g) | Total electrical energy  required (kWh/g) | Solar energy  required (kWh/g) |
| --- | --- | --- | --- |
| KL-HTC-800-conv-1000 | 5.36 | 9.3 | - |
| KL-HTC-800-conv-1400 | 4.98 | 11.83 | - |
| KL-HTC-800-conv-1800 | 5.91 | 17.66 | - |
| KL-HTC-800-solar-1000 | 5.44 | 0.70 | 0.79 |
| KL-HTC-800-solar-1400 | 5.41 | 0.70 | 1.76 |
| KL-HTC-800-solar-1800 | 5.74 | 0.70 | 3.38 |

Overall, the solar carbonization is less energy demanding than electrical carbonization. The initial mass required to produce 1 g of graphitic biocarbon is almost similar across all high-temperature treatment routes proposed. It should be noted that the main electrical input for the solar samples is due to the production of the initial precursor (hydrothermal carbonization and pyrolysis) which remain energy-efficient steps compared to the high-temperature conventional carbonization.
